# Nutrition literacy and eating habits in children from food-secure versus food-insecure households: A cross-sectional study

**DOI:** 10.1097/MD.0000000000039812

**Published:** 2024-09-27

**Authors:** Maral Hashemzadeh, Masoumeh Akhlaghi, Marzieh Akbarzadeh, Kiana Nabizadeh, Hamid Heidarian Miri, Asma Kazemi

**Affiliations:** a Department of Community Nutrition, School of Nutrition and Food Sciences, Shiraz University of Medical Sciences, Shiraz, Iran; b School of Food and Nutritional Sciences, University College Cork, Cork, Ireland; c Nutrition Research Center, Shiraz University of Medical Sciences, Shiraz, Iran.

**Keywords:** child, diet, food, and nutrition, dietary habits, food insecurity, knowledge

## Abstract

This study aimed to explore whether maternal food and nutrition literacy (FNL) can mitigate negative effects of food insecurity on children’s eating habits and assist food-insecure households in making better nutrition choices with limited resources available. A cross-sectional study was conducted on children aged 6 to 12 years and their mothers. FNL was assessed using a validated Food and Nutrition Literacy Assessment Tool, and household food security was evaluated with the Household Food Insecurity Access Scale. Eating habits were measured through a structured questionnaire that focused on various dietary habits. Logistic regression was used to determine the associations between maternal FNL, food insecurity status, and children’s eating habits. A total of 327 children–mothers, comprising 159 food-secure and 168 food-insecure households, were evaluated. Maternal FNL was inversely associated with consumption of fast-foods and fried foods, and skipping breakfast in both food-secure and food-insecure participants. However, only food-secure participants showed an inverse association between FNL and the habit of eating out. Both groups indicated lower sweet consumption associated with higher FNL. Maternal FNL was inversely associated with lower dairy and nut intake in food-secure group and lower fruit and vegetable consumption in food-insecure participants. This study suggests that in conditions of food insecurity FNL may reduce the negative impact of food insecurity on children’s nutrition choices and food habits.

## 1. Introduction

Eating habits are critical determinants of health, influencing growth, development, and the risk of chronic diseases in children.^[1–3]^ Childhood is a crucial period for the development of lifelong eating habits.^[4]^ Childhood eating habits can have significant and lasting consequences on physical, emotional, and social development.^[5]^ Healthy eating habits reduce the risk of obesity^[6]^ and chronic diseases such as type 2 diabetes, cardiovascular disease, and certain cancers,^[7]^ improve nutritional status, growth and development,^[8]^ enhance cognitive function, promote better mood and emotional stability,^[9]^ and improve academic performance and social interactions,^[10]^ all of these can ultimately enhance future educational and economic opportunities. Thus, understanding the factors that shape children’s eating habits is essential for promoting better health outcomes.

Nutrition knowledge, or food and nutrition literacy (FNL), plays a pivotal role in shaping eating habits.^[11]^ It encompasses the understanding of nutritional information, dietary guidelines, and the ability to make informed food choices.^[12]^ Studies have demonstrated that higher levels of FNL among caregivers, particularly mothers, are associated with healthier dietary practices in children.^[13]^ This relationship suggests that improving FNL can empower families to conform healthier eating habits, thereby enhancing children’s health and well-being.

Food insecurity, defined as the lack of consistent access to sufficient food for an active, healthy life,^[14]^ poses significant challenges to healthy eating habits.^[15,16]^ Children from food-insecure households often experience limited access to nutritious foods, leading to a reliance on cheaper, less healthy options.^[17,18]^ This reliance can contribute to poor dietary quality and increased risk of obesity and other health issues.^[18]^ Additionally, food insecurity is associated with irregular meal patterns and higher consumption of fast and fried food and unhealthy cooking methods,^[15]^ further exacerbating health disparities among vulnerable populations.^[19]^

Although multiple studies have investigated the relationship between food insecurity and eating habits as well FNL and eating habits, there is a literature gap on the relationship between eating habits and food and FNL with regard to the food insecurity. One uncertainty lies in the extent to which maternal FNL can mitigate the adverse effects of food insecurity on children’s dietary choices. There are studies that investigated the relationship between eating habits and food and nutrition literacy with regard to socioeconomic conditions.^[20–22]^ Conflicting findings exist in the literature regarding whether higher FNL consistently leads to healthier eating habits across different socioeconomic contexts.^[20–22]^ Some studies suggest that while FNL is beneficial, its impact may be diminished in lower socioeconomic conditions, creating a paradox where knowledge does not translate into practice due to external constraints such as affordability and availability of nutritious options,^[21,22]^ while other studies indicated that nutrition knowledge is likely to reduce socioeconomic differences in compliance with dietary guidelines.^[20]^

Understanding the dynamics between FNL, food security, and dietary habits is crucial for both healthy individuals and patients, particularly those at risk of diet-related diseases. Evidence from studies indicates that improved FNL can lead to better dietary practices, which are essential for preventing obesity, diabetes, and other chronic conditions.^[23,24]^ For healthy individuals, this knowledge can promote lifelong healthy eating patterns, while for patients, particularly those with existing health issues, it can inform tailored interventions that address both nutritional knowledge and practical food access challenges. This research underscores the need for public health strategies that enhance FNL as a means to improve dietary behaviors and health outcomes across varying levels of food security.^[25]^

This study aimed to explore whether maternal FNL can mitigate the negative effects of food insecurity on children eating habits and assist the food-insecure households in making better choices with the limited available resources.

## 2. Methods

### 2.1. Study design

This study employed a cross-sectional design to assess the relationship between maternal FNL and eating habits among children from food-secure and food-insecure households. The study was conducted in elementary schools of Shiraz during 2023. The study protocol was approved by the Research Ethics Committee of Shiraz University of Medical Sciences (IR. SUMS.SCHEANUT. REC.1402.019). All data collected were kept confidential, and participants were assigned unique identifiers to ensure their anonymity.

### 2.2. Study population and sample size estimation

Participants included children aged 6 to 12 years and their mothers. The inclusion criteria for children were: (1) being enrolled in selected schools, as described below, and (2) having parental consent. The inclusion criteria for mothers were (1) being aged between 20 and 55 years, and (2) having no specific medical conditions, such as cancer, chronic metabolic diseases, liver or kidney disorders, thyroid diseases, cognitive problems, pregnancy, or breastfeeding.

The sample size was determined using Power Analysis & Sample Size (PASS) software. An estimated sample size of 330 was calculated, considering a type 1 error rate of 5%, a power of 80%, and prevalence rates of 56% for food insecurity and 14% for poor FNL. This estimation aimed to identify the relationship between these factors through logistic regression, based on an adjusted odd ratio (OR) of 2.89 reported in the study by Khorramrouz et al.^[26]^ To account for a potential dropout of 20 participants, the final sample size was set at 350.

The participants were recruited from 9 elementary schools. Schools were selected equally from 3 socioeconomic categories: affluent, semi-affluent, and deprived. Thirty-five students were selected from each school. Stratified random sampling was used to select the students, while cluster random sampling was employed to select the schools. Thus, the sampling frame for this study was multi-stage, with socioeconomic categories serving as the strata and schools as the clusters.

### 2.3. Data collection

#### 2.3.1. Assessment of FNL

FNL was assessed using the Food and Nutrition Literacy Assessment Tool by face-to-face interviews. Food and Nutrition Literacy Assessment Tool is a 60-items questionnaire that has been validated in Iran.^[27]^ It is composed of 2 main domains including knowledge and practical skills related to food and nutrition and 6 dimensions including advocacy, functional skills, food and nutrition knowledge, interactive skills, critical analysis of information, and food label reading competence. The knowledge domain and food label reading skills are evaluated by 30 binary questions, while the other skill domains (excluding food label reading) are assessed through 30 Likert-type statements. The overall FNL score, as well as the scores for each dimension, ranged from 0 to 100. The total scores were linearly converted to fit within this range, with higher scores indicating a higher level of FNL. Scores below 60 are classified as poor, while scores above 60 indicate adequate or good FNL.

#### 2.3.2. Household food security assessment

The researchers evaluated household food insecurity using the Household Food Insecurity Access Scale (HFIAS), a validated tool designed to assess food insecurity in developing countries.^[28]^ The HFIAS questionnaire consists of 9 items that have been translated and validated for use in the Iranian population. Each question has 4 response options corresponding to the frequency of experiencing that situation in the past month: 0 = never, 1 = rarely (once or twice), 2 = sometimes (3–10 times), and 3 = often (more than 10 times). The HFIAS questions cover 3 main aspects of food insecurity access: (1) concerns and uncertainty about household food supply; (2) inadequate quality (including variety and food preferences); and (3) insufficient food intake and its physical consequences. The HFIAS scale ranges from 0 to 27 and classifies food insecurity into 4 levels: food-secure (0–1), mildly food-insecure (2–8), moderately food-insecure (9–14), and severely food-insecure (15–27).

### 2.4. Eating habits

Eating habits were evaluated through a structured questionnaire that have been previously used in Iranian women.^[15]^ The questionnaire included questions on the frequency of several dietary habits, including eating breakfast, fast-food, fried food, and take-away food or eating out. The questionnaire also asked about snack preferences, including fruits, vegetables, dairy products, nuts, and sweets. Additionally, it explored common cooking methods, particularly techniques used for preparing rice that are specific to typical Iranian cuisine.

### 2.5. Anthropometric measurement

Height and weight were measured for both mothers and children. Weight was recorded using a digital scale (Glamor BS-801 Hitachi China) with an accuracy of 100 grams, with participants dressed in minimal clothing and without shoes. Height was measured using a tape measure sensitive to 0.1 cm, ensuring that participants stood barefoot against a wall with their shoulders, hips, and heels in contact with it.

### 2.6. Statistical analysis

Data were analyzed using STATA version 17. The normal distribution of quantitative variables was evaluated using 3 approaches: (1) visually, by inspecting a histogram; (2) statistically, through the Shapiro–Wilk test; and (3) by evaluating measures of central dispersion. Chi-square tests were employed to compare categorical variables, while *t* tests were used for continuous variables.

Multiple logistic regression was conducted to assess the associations between children’s eating habits and maternal FNL, as well as, food insecurity. Unadjusted and adjusted OR with 95% confidence intervals (CI) were calculated. Potential confounders included in the adjusted model were sex and age of children, age and education of mothers, household size, and income.

## 3. Results

A total of 350 mother/child pairs participated in the present study. However, 23 participants were excluded for not meeting the inclusion or exclusion criteria, resulting in a final sample of 327 mothers included in the analysis, with 159 from food-secure and 168 from food-insecure households. The demographic characteristics of the participants are summarized in Table 1. The sample included an approximately balanced gender distribution, with 55% of participants identifying as male and 45% as female. The mean age of the children and mothers was 9.0 (standard deviation = 1.72) and 33.08 (5.08) years, respectively. There were no significant differences in children age, sex, body mass index (BMI), and BMI for age z-score between food-secure and -insecure groups, but mothers’ age and BMI were higher in food-insecure group. When comparing participants based on maternal FNL, there were no significant differences in age or sex among the children. However, children whose mothers had higher FNL exhibited higher BMI and BMI for age z-score. Regarding the eating habits, FNL groups differ in all food habits except method of cooking rice. Similarly, food insecurity groups differed in all eating habits, except for the cooking method, and rice cooking method.

**Table 1:** Characteristics of mothers and children according to household food security and maternal food and nutrition literacy.

Variables	Total (n = 327)	Food-secure (n = 159)	Food-insecure (n = 168)	*P* value	Poor FNL (n = 187)	Good FNL (n = 140)	*P* value
Mother Age, y	38.1 ± 5.1	37.2 ± 4.6	38.8 ± 5.3	.003	37 ± 5	39 ± 5	<.001
Mother BMI, kg/m^2^	26.7 ± 5.5	24.9 ± 4.5	28.1 ± 5.8	<.001	30.13 ± 5.23	24.13 ± 4.12	<.001
Child age, y	9.0 ± 1.8	8.9 ± 1.7	9.0 ± 1.8	.63	9 ± 2	9 ± 2	.63
Child BMI, kg/m^2^	18.0 ± 4.0	17.5 ± 3.3	18.4 ± 4.4	.060	17.03 ± 3.4	19.33 ± 4.4	<.001
Child BMI-z-score	0.41 ± 1.8	0.2 ± 1.5	0.5 ± 1.8	.25	0.08 ± 1.6	0.84 ± 1.9	<.001
Child sex, Girl n (%)	180 (55.0)	92 (57.9)	88 (52.4)	.32	106 (56.7)	74 (52.9)	.49
FNLAT-SCORE	61.4 ± 13.7	66.4 ± 11.8	56.5 ± 13.6	<.001	71.13 ± 7.3	48.33 ± 8.4	<.001
HFIAS-SCORE	2.8 ± 3.9	0.1 ± 0.3	5.2 ± 4.2	<.001	2 ± 3	4 ± 5	<.001
Food insecurity status, n (%)							
Secure	159 (48.6)	–	–		42 (30.0)	117 (62.57)	<.001
Insecure	168 (51.4)	–	–		98 (70.0)	70 (1.07)	
Mother education, n (%)							
Lower than university	209 (64.11)	70 (44.3)	139 (82.7)	<.001	116 (82.9)	93 (50.0)	<.001
University	117 (35.89)	88 (55.7)	29 (17.3)		24 (17.1)	93 (50.0)	
Mother job, n (%)							
House wife	266 (81.60)	117 (74)	149 (88.7)	<.001	141 (75.8)	125 (89.3)	.005
Employee	56 (17.18)	40 (25.3)	16 (9.5)		43 (23.1)	13 (9.3)	
Labor	4 (1.23)	1 (0.6)	3 (1.8)		2 (1.1)	2 (1.4)	
Fast-food							
0–3 times per month	241 (74.6)	128 (82.1)	113 (67.7)	.003	173 (94.0)	68 (48.9)	<.001
≥4 times per month	82 (25.4)	28 (17.9)	54 (32.3)		11 (6.0)	71 (51.1)	
Eating out or takeaway							
0–3 times per month	284 (88.20)	127 (81.9)	157 (94.0)	<.001	116 (83.5)	168 (91.8)	.02
≥4 times per month	38 (11.80)	28 (18.1)	10 (6.0)		23 (16.5)	15 (8.2)	
Fried foods per week							
0–5 times per month	215 (67.0)	114 (73.1)	101 (61.2)	<.001	158 (86.3)	57 (41.3)	<.001
≥6 times per month	106 (33.0)	42 (26.9)	64 (38.8)		25 (13.7)	81 (58.7)	
Breakfast							
0–4 times per week	203 (62.5)	32 (20.4)	90 (53.6)	<.001	26 (14.0)	96 (69.1)	<.001
5–7 times per week	122 (37.5)	125 (79.6)	78 (46.4)		160 (86.0)	43 (30.9)	
Cooking method							
Boiling or grilling	137 (42.41)	70 (44.9)	67 (40.1)	.39	26 (18.8)	111 (60.0)	<.001
Frying	186 (57.59)	86 (55.1)	100 (59.9)		112 (81.2)	74 (40.0)	
Cooking rice							
Drain	192 (59.3)	65 (41.4)	67 (40.1)	.81	69 (37.3)	63 (45.3)	.15
Stream	132 (40.7)	92 (58.6)	100 (59.9)		116 (62.7)	76 (54.7)	
Foods as snacks (Yes)							
Fruit and vegetables	109 (33.9)	135 (86.0)	78 (47.3)	<.001	150 (81.1)	63 (46.0)	<.001
Dairy	275 (85.4)	40 (25.5)	7 (4.2)	<.001	43 (23.2)	4 (2.9)	<.001
Nuts	262 (81.4)	56 (35.7)	4 (2.4)	<.001	53 (28.6)	7 (5.1)	<.001
Sweets	156 (48.4)	57 (36.3)	99 (60.0)	<.001	56 (30.3)	100 (73.0)	<.001

Categories were reported as number or percentage of subjects. Continuous variables were reported by means and standard deviation.

Independent *t* test was used for quantitative variables and χ^2^ test was used for qualitative variables.

BMI = body mass index, FNL = food & nutrition literacy, FNLAT = Food and Nutrition Literacy Assessment Tool, HFIAS = Household Food Insecurity Access Scale.

Among unhealthy eating habits, food insecurity was associated with a higher risk of fast-food consumption (OR: 1.99, 95% CI: 1.05–3.772) and fewer days of breakfast intake (OR: 2.52, 95% CI: 1.41–4.55) (Table 2). Additionally, food insecurity was associated with a higher risk of consuming more sweets and fewer fruit and vegetables, dairy products, and nuts as snacks.

**Table 2:** The relationship between household food insecurity (insecure vs secure) and children eating habits.

Food behaviors	Categories	OR (95% CI), crude	OR (95% CI), adjusted*
Fast-food consumption	≥4 vs <4 time/month	2.18 (1.30–3.68)	1.99 (1.05–3.77)
Eating out or takeaway	≥4 vs <4 time/month	0.29 (0.14–0.62)	0.40 (.16–1.01)
Fried food	≥6 vs <6 time/week	1.72 (1.07–2.76)	0.99 (0.54–1.82)
Cooking method	Frying vs boiling/grilling	1.21 (0.78–1.89)	0.70 (0.39–1.25)
Breakfast intake	≤4 vs >4-time week	4.51 (2.75–7.38)	2.52 (1.41–4.55)
Time of breakfast	8:01–10:0 vs 6:0–8:0	0.98 (0.47–2.0)	0.76 (0.30–1.94)
Time of lunch	13:31–15:0 vs 12:0–13:30	0.46 (0.25–0.83)	0.56 (0.27–1.18)
Time of dinner	20:31–22:0 vs 19:0–20:30	0.72 (0.39–1.31)	0.80 (0.38–1.69)
Fruit/vegetable intake	No vs yes	6.84 (3.97–11.80)	3.36 (1.76–6.40)
Dairy intake	No vs yes	7.72 (3.34–17.83)	8.04 (2.94–21.98)
Nut intake	No vs yes	22.32 (7.85–63.42)	17.82 (4.91–64.71)
Sweets intake	Yes vs no	2.63 (1.68–4.13)	2.04 (1.17–3.56)
Cooking rice method	Draining vs steaming	0.95 (0.61–1.48)	0.61 (0.34–1.10)

* Adjusted for age (mothers and children), children gender, mothers’ education, family size, and income.

FNL was associated with a lower risk of frequent fried (OR: 0.12, 95% CI: 0.07–0.22) and fast-food consumption (OR: 0.05, 95% CI: 0.02–0.10), eating out or ordering take-away (OR: 0.17, 95% CI: 0.07–0.43), and using frying cooking methods (OR: 0.20, 95% CI: 0.11–0.35), as well as lower risk of less frequent breakfast consumption (OR: 0.08, 95% CI: 0.04–0.15) (Table 3). Moreover, FNL was associated with a lower risk of consuming more sweets and fewer fruits, vegetables, dairy products, and nuts as snacks.

**Table 3:** The relationship between maternal food and nutrition literacy (FNL) (have FNL vs have not FNL) and children eating habits.

Food behaviors	Categories	OR (95% CI), crude	OR (95% CI), adjusted*
Fast-food consumption	≥4 vs <4 time/ month	0.06 (0.03–0.12)	0.05 (0.02–0.10)
Eating out or takeaway	≥4 vs <4 time/ month	0.45 (0.23–0.90)	0.17 (0.07–0.43)
Fried food	≥6 vs <6 time/ week	0.11 (0.06–0.19)	0.12 (0.07–0.22)
Cooking method	Frying vs boiling/grilling	0.15 (0.09–0.26)	0.20 (0.11–0.35)
Breakfast intake	≤4 vs >4-time week	0.07 (0.04–0.13)	0.08 (0.04–0.15)
Time of breakfast	8:01–10:0 vs 6:0–8:0	0.70 (0.31–1.58)	0.92 (0.36–2.37)
Time of lunch	13:31–15:0 vs 12:0–13:30	1.19 (0.62–2.26)	0.82 (0.39–1.73)
Time of dinner	20:31–22:0 vs 19:0–20:30	0.81 (0.42–1.56)	0.72 (0.35–1.51)
Fruit/vegetable intake	No vs yes	1.99 (0.12–0.33)	0.24 (0.13–0.43)
Dairy intake	No vs yes	0.10 (0.03–0.28)	0.14 (0.05–0.43)
Nut intake	No vs yes	0.13 (0.06–0.31)	0.19 (0.07–0.50)
Sweets intake	Yes vs no	0.16 (0.10–0.26)	0.14 (0.08–0.25)
Cooking rice method	Draining vs steaming	0.72 (0.46–1.12)	0.76 (0.45–1.29)

* Adjusted for age (mothers and children), children gender, mothers’ education, family size, and income.

In both food-secure and food-insecure groups, higher maternal FNL was associated with a lower risk of frequent fast-food consumption (OR: 0.03, 95% CI: 0.01–0.10 in food-secure; OR: 0.05, 95% CI: 0.01–0.16 in food-insecure), eating fried foods (OR: 0.09, 95% CI: 0.03–0.23 in food-secure; OR: 0.13, 95% CI: 0.05–0.33 in food-insecure), and using frying methods (OR: 0.13, 95% CI: 0.05–0.38 in food-secure; OR: 0.18, 95% CI: 0.08–0.39 in food-insecure), as well as less frequent breakfast consumption (OR: 0.08, 95% CI: 0.03–0.20 in food-secure; OR: 0.09, 95% CI: 0.04–0.21 in food-insecure) (Table 4). However, a lower risk of eating out or ordering take-out was observed only in the food-secure group. Regarding snack consumption, higher maternal FNL was associated with a lower risk of excessive sweet consumption in both food-secure and food-insecure groups. However, the risk of reduced dairy product and nut intake was lower only in the food-secure group. Conversely, higher FNL was associated with a lower risk of reduced fruit and vegetable consumption solely in the food-insecure group.

**Table 4:** Relationships between maternal food and nutrition literacy and children eating habits among subgroups of food-secure and -insecure households.

Food behaviors	Categories	Food insecurity status	OR (95%CI), crude	OR (95%CI), adjusted*
Fast-food consumption	≥4 vs <4 time/month	Food-secure	0.05 (0.03–0.13)	0.03 (0.01–0.10)
		Food-insecure	0.08 (0.03–0.21)	0.05 (0.01–0.16)
Eating out or takeaway	≥4 vs <4 time/month	Food-secure	0.27 (0.11–0.63)	0.15 (0.05–0.44)
		Food-insecure	0.15 (0.02–1.18)	0.11 (0.01–1.17)
Fried food	≥6 vs <6 time/week	Food-secure	0.11 (0.05–0.25)	0.09 (0.03–0.23)
		Food-insecure	0.10 (0.04–0.23)	0.13 (0.05–0.33)
Cooking method	Frying vs boiling/grilling	Food-secure	0.11 (0.04–0.31)	0.13 (0.05–0.38)
		Food-insecure	0.14 (0.07–0.27)	0.18 (0.08–0.39)
Breakfast intake	≤4 vs >4-time week	Food-secure	0.10 (0.04–0.24)	0.08 (0.03–0.20)
		Food-insecure	0.08 (0.04–0.17)	0.09 (0.04–0.21)
Time of breakfast	8:0–10:0 vs 6:0–8:0	Food-secure	0.90 (0.26–3.08)	1.17 (0.26–5.18)
		Food-insecure	0.54 (0.17–1.71)	0.61 (0.16–2.37)
Time of lunch	13:31–15:0 vs 12:0–13:30	Food-secure	0.91 (0.34–2.41)	0.63 (0.20–2.03)
		Food-insecure	1.06 (0.41–2.70)	0.90 (0.30–2.66)
Time of dinner	20:31–22:0 vs 19:0–20:30	Food-secure	0.96 (0.37–2.49)	0.92 (0.31–2.74)
		Food-insecure	0.57 (0.22–1.47)	0.50 (0.17–1.53)
Fruit/vegetable intake	No vs yes	Food-secure	0.57 (0.22–1.47)	0.44 (0.15–1.25)
		Food-insecure	0.20 (0.10–0.39)	0.23 (0.10–0.50)
Dairy intake	No vs yes	Food-secure	0.17 (0.05–0.58)	0.18 (0.05–0.66)
		Food-insecure	0.11 (0.01–0.94)	0.17 (0.02–1.63)
Nut intake	No vs yes	Food-secure	0.18 (0.06–0.48)	0.14 (0.04–0.45)
		Food-insecure	0.71 (0.10–5.19)	0.48 (0.02–10.41)
Sweet intake	Yes vs no	Food-secure	0.18 (0.08–0.39)	0.13 (0.05–0.31)
		Food-insecure	0.19 (0.10–0.37)	0.18 (0.08–0.39)
Cooking rice method	Draining vs steaming	Food-secure	0.76 (0.37–1.56)	0.57 (0.25–1.30)
		Food-insecure	0.61 (0.32–1.16)	0.72 (0.35–1.52)

* Adjusted for age (mothers and children), children gender, mothers’ education, and income.

## 4. Discussion

This study aimed to examine the relationship between maternal FNL and eating habits among children from food-secure and food-insecure households. Our findings indicate that maternal FNL is significantly associated with children’s eating habits, with notable differences observed between the status of household food security.

A critical finding of this study is the positive association between higher maternal FNL and healthier eating habits among children. Children of mothers with higher FNL demonstrated a lower risk of unhealthy eating habits, including frequent fried and fast-food consumption, eating out, missing breakfast, and the intake of sweets. This suggests that maternal nutritional literacy plays a pivotal role in shaping children’s dietary practices, potentially through improved food choices and cooking methods. These results are consistent with previous studies that have highlighted the importance of nutritional education in promoting healthier eating habits among families.^[13,29]^

Yabanci et al (2014) conducted a cross-sectional study on 302 mothers of the students in Turkey.^[29]^ They indicated that mothers with higher FNL provided their children with more vegetables, fruits, and legumes, while limiting sugary drinks like soda, juice, and fast-food compared to mothers with lower FNL levels. Additionally, mothers with higher FNL were more likely to avoid feeding their children foods containing artificial ingredients. Another study on 1043 Chinese school-age children, showed that the dietary literacy of those providing food may significantly impact children’s health and eating habits.^[13]^ Conversely, in a systematic review of observational studies on children and adolescents (5–19 years),^[30]^ 4 studies indicated no significant association between overall nutrition knowledge and dietary practices, and only 2 identified a significant association, with one of them revealing a weak link, suggesting that nutrition knowledge alone may be insufficient to promote healthy eating habits. The differences between the results of this systematic review and our study, as well as the other studies mentioned, may stem from the fact that this review assessed the nutrition knowledge of children and adolescents, whereas our study and the others focused on the nutrition knowledge of parents or guardians.

The results of present study indicated that children from food-insecure households exhibited poorer eating habits compared to their counterparts in food-secure households. Specifically, food insecurity was associated with increased fast-food consumption, fewer days of breakfast intake, and a higher intake of sweets, while fruit, vegetable, dairy, and nut consumption was notably lower. These findings are consistent with previous research, such as that by Dobuis et al (2023), which found that being at high risk (compared with low risk) of food insecurity in childhood up to adolescence was associated with consuming higher quantities of sugar-sweetened beverages, non-whole-grain cereal products, and processed meat, with skipping breakfast, with eating meals prepared out of home, once reaching young adulthood.^[31]^ In another study on 4001 children aged 5 to 11 years and their parents in Lebanon, severe food insecurity worsened eating habits of children during the COVID-19 pandemic.^[32]^ In a study on adult females, food-insecure individuals were more likely to exhibit unhealthy dietary habits, such as skipping breakfast, eating fewer snacks, consuming more fast-food and fried dishes, and employing unhealthy cooking techniques, compared to food-secure participants.^[15]^

Interestingly, while higher maternal FNL was associated with improved eating habits in both food-secure and food-insecure groups, the association was more pronounced in the food-secure group, particularly regarding the consumption of dairy products and nuts. This suggests that while nutrition literacy can mitigate some negative eating habits in food-insecure households, structural barriers related to food access may still hinder optimal dietary practices. To the best of our knowledge, no study has assessed the relationship between FNL and children eating habit in the context of food insecurity.

## 5. Implications for interventions

The findings of this study have important implications for interventions aimed at improving children nutritional practice. Programs that enhance maternal nutritional literacy could be particularly beneficial in both food-secure and food-insecure contexts. Nonetheless, simply increasing nutrition literacy may not be sufficient to improve dietary practices in food-insecure environments. Public health initiatives should therefore focus on creating comprehensive nutrition education programs that not only enhance knowledge but also address the structural barriers that limit access to healthy foods in food-insecure households. By integrating strategies to improve food availability and affordability alongside nutrition education, public health efforts can empower families to make healthier food choices, ultimately promoting better dietary outcomes for children across varying socioeconomic backgrounds.

## 6. Limitations and future research

While this study provides valuable insights, it is essential to acknowledge its limitations. The cross-sectional design limits causal inferences, and the reliance on self-reported data may introduce bias. The small sample size is another limitation that should be considered. Another limitation is that we didn’t assess dietary intake of children. Furthermore, we did not assess psychological factors like depression and stress, which could have influenced eating habits. Future research with a larger sample size is recommended to validate these results. Additionally, future research should consider longitudinal studies or randomized controlled trial to better understand the causal relationships between FNL, food insecurity, and eating habits. Moreover, qualitative research could provide deeper insights into the challenges faced by families and the strategies they employ to navigate food choices. Understanding these dynamics can inform the development of tailored interventions that address both nutrition literacy and food access.

## 7. Conclusion

In conclusion, this study highlights the significant relationship between maternal food and nutrition literacy and children’s eating habits, particularly in the context of food security. Enhancing nutrition literacy has the potential to improve dietary practices among children, especially those from food-insecure households. By addressing both nutrition education and food access, we can work towards fostering healthier eating habits and improving overall health outcomes for vulnerable populations.

## Author contributions

**Conceptualization:** Maral Hashemzadeh, Asma Kazemi.

**Data curation:** Hamid Heidarian Miri.

**Formal analysis:** Hamid Heidarian Miri.

**Investigation:** Maral Hashemzadeh, Kiana Nabizadeh.

**Project administration:** Asma Kazemi.

**Supervision:** Asma Kazemi.

**Writing – original draft:** Asma Kazemi.

**Writing – review & editing:** Maral Hashemzadeh, Masoumeh Akhlaghi, Marzieh Akbarzadeh.
